# Impact of forest vegetation on soil characteristics: a correlation between soil biological and physico-chemical properties

**DOI:** 10.1007/s13205-016-0510-y

**Published:** 2016-09-01

**Authors:** L. R. Chandra, S. Gupta, V. Pande, N. Singh

**Affiliations:** 1 Plant Ecology and Environmental Science Division, CSIR-National Botanical Research Institute, Rana Pratap Marg, Lucknow, Uttar Pradesh 226 001 India; 2 Department of Biotechnology, Bhimtal Campus, Kumaun University, Nainital, Uttarakhand 263136 India

**Keywords:** Soil microbial biomass, Soil quality, Forest ecosystem, Nutrient cycle, Enzyme activity

## Abstract

Temperate and dry deciduous forest covers major portion of terrestrial ecosystem in India. The two forest types with different dominant tree species differ in litter quality and root exudates, thereby exerting species-specific impact on soil properties and microbial activity. This study aims to examine the influence of forest type or dominant tree species on soil physico-chemical properties and its relationship with microbial characters in temperate and dry deciduous forest types. We assessed soil physico-chemical properties among five different sites located within the selected forest stand covered by different dominant species. The soil microbial biomass carbon (MBC), nitrogen (MBN) and phosphorous (MBP) were recorded high in oak soil, i.e., the MBC/TOC ratio was significantly higher in dry deciduous forest. Basal respiration was recorded highest at oak-mixed soil while qCO_2_ was comparatively high in oak soil. Temperate forest displayed the highest MBC/MBN ratio, while dry deciduous forest had the highest MBC/MBP ratio. Moreover, the MBN/TN ratio was found high in dry deciduous forest, whereas MBP/TP ratio was high in temperate forest. Additionally, the enzyme activities were significantly higher in an oak-mixed soil among all the sites. The results displayed that the soil microbial characters and soil physico-chemical uniqueness are interrelated, and were significantly influenced by specific forest type and climatic variables.

## Introduction

Soil microorganisms are important components of terrestrial ecosystems because they play an important role in intrinsic phenomena like nutrient cycle and ecosystem functioning which directly involve in maintaining soil fertility and its structure. Soil microbial biomass formation is the living portion of soil organic matter, responsible for the decomposition and mineralization of the organic matter fraction that acts both as a sink and a source of nutrient which become available during the turnover of microbial biomass (Chen et al. 2005). Therefore, the information regarding the factors that control the soil microorganism and microbial biomass is fundamental to a sustainable environmental system. The accessibility and uptake of nutrients is directly proportional to the living component of the given soil niche. Thus, soil microbial processes strongly regulate the stability, fertility and functioning of ecosystems and used to assess the soil excellence among different vegetations (Fierer et al. 2003).

Due to various effects, the assessment of soil quality is relatively complicated. A specific combination of physico-chemical and biological factors is required for maintaining the soil strength. Understanding of these features supports in knowing the soil health. The major portion of terrestrial ecosystems is covered by forest land. Soil microbes regulate the decomposition rate, organic matter content of soil and the overall biogeochemical processes that govern the productivity of forest ecosystems (Six et al. 2004; Noguez et al. 2008) and in turn, the soil physico-chemical properties of the given forest type control the microbial biomass as well as their activity. Forest stands covered with different tree species; differ in litter quality and root exudates. This difference ultimately generates a divergence in soil properties and may influence the soil microbial community.

Natural forests that occur all over the world are formed through natural regeneration following stand-replacing disturbances by anthropogenic activities or by extreme natural events (Yang et al. 2010; Wang and Yang 2007). Approximately 30 % of total land area is covered by forest (boreal, temperate and tropical forest) (Holden and Treseder 2013). These forests are the source of global terrestrial carbon in which temperate forest ecosystem plays a major role in carbon sequestration from increasing atmospheric carbon dioxide, as it covers the major portion of terrestrial land (Myneni et al. 2001). It has been predicted that naturally regenerated forest stands are generally superior to plantations in nutrient cycling and soil quality (Burton et al. 2007), but some conflicting experimental results suggest that variations in soil microbial biomass probably depend on the specific forest ecosystems and climatic factors.

Various studies have been given to understand interrelationship between the tree species, soil microbial and physico-chemical properties of forest soil. Further, studies undertaken on varied forest soils confirm that various biotic and abiotic factors influence the microbial biomass, composition and activity (Landesman and Dighton 2011). Still, the information is limited as several factors control this interrelationship in the forest ecosystem. Consequently, the present study has been undertaken in different natural forest stands from dry deciduous and temperate region of North-India that contains a wide range of biodiversity. The main aim of the study is to hypothesize the impact of the specific forest type of biological properties that mainly include the difference in soil microbial biomass, respiration and soil enzymes activity, and interpret the correlation between soil biological and physico-chemical characteristics.

## Materials and methods

### Study sites, soil sampling and pretreatment

**Fig. 1:**
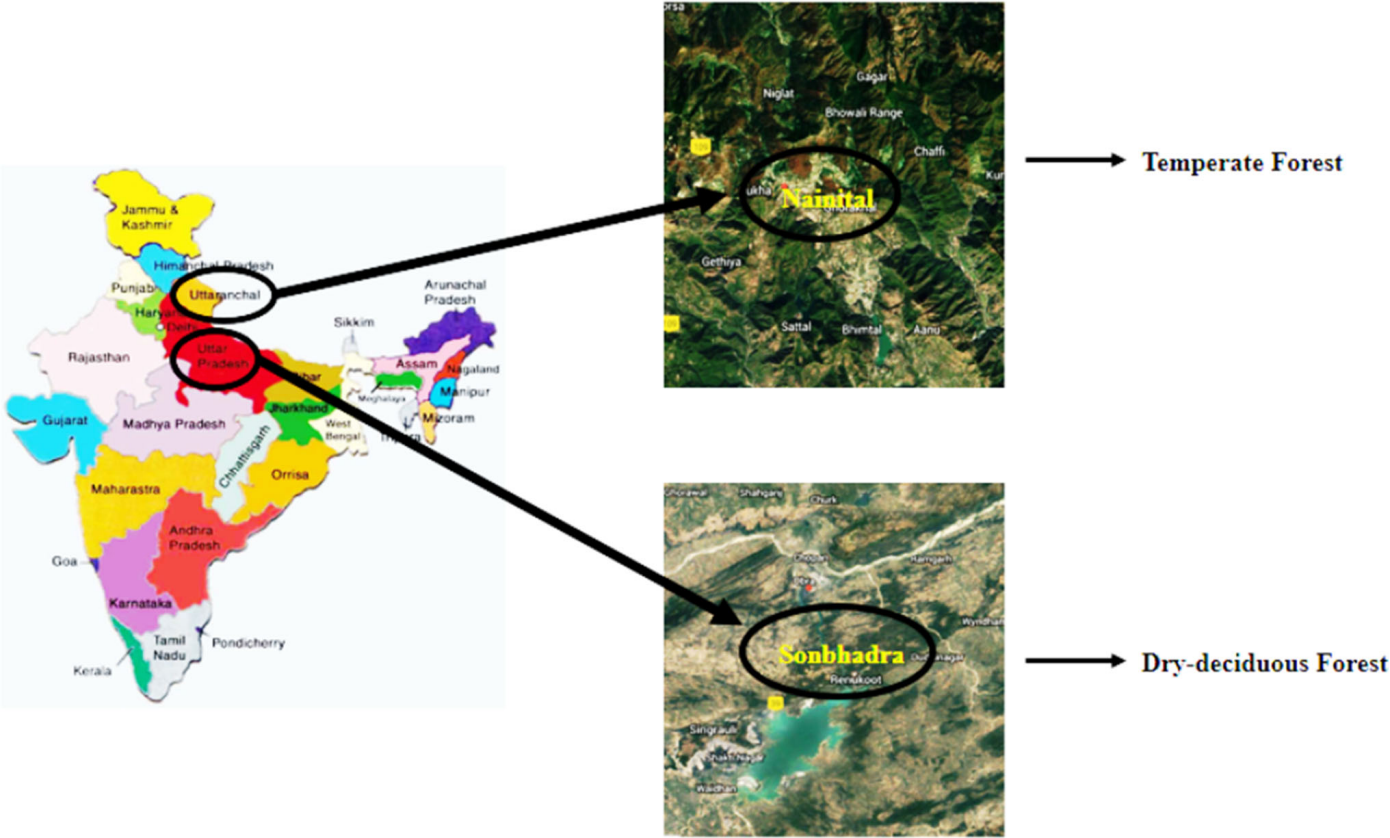
Selected area and forest types. Source: Google Map

The study has been undertaken in two different natural forest types of India. The selected forest types were located in Nainital and Sonbhadra district of Uttarakand and Uttar Pradesh states, respectively (Fig. 1). The two forest types are classified into dry deciduous and temperate forest. The *Shorea robusta* (sal) is the dominant species of Sonbhadra forest, dry deciduous in nature and is considered as one of the best Sal forests of India, followed by *Diospyros melanoxylon*, *Lagerstroemia parviflora*, *Anogeissus latifolia*, *Bridelia retusa*, *Hardwickia binata*, *Mallotus philippensis*, *Buchanania lanzan* and *Terminalia elliptica* as co-dominant species. Similarly, temperate forest sites of Nainital district comprise of three dominant plant communities, one dominated by *Pinus roxburghii* (pine) and other two by *Quercus floribunda* and *Quercus leucotrichophora* (oak), the other co-dominant plant communities are *Quercus semecarpifolia*, *Rhododendron arboretum*, *Myrica esculenta*, *Berberis asiatica* and *Urtica parviflora.* All the stands share different forest age and soil type. A total of five stands were selected among the two forest types. The two stands of dry deciduous forest are selected on the basis of vegetation covered. One stand is dominated by sal (*Shorea robusta*) vegetation while other stand is sal-mixed vegetative stand. Similarly, the three selected stands located in temperate region are dominated by pine (*Pinus roxburghii*), oak (*Quercus leucotrichophora*) and oak-mixed vegetation, respectively. During the study period, the minimum temperature ranged from 6 to 29 °C and maximum temperature varied from 28 to 42 °C in the forest of the dry deciduous region. Whereas, the minimum temperature in the forest of temperate region is recorded as −3 to 12 °C and maximum range is 18–30 °C. The minimum and maximum temperature was recorded during the month of January and June, respectively. Additionally, the recorded average annual rainfall for dry deciduous and temperate forest was 73.1 and 151.9 mm, respectively. The physiographic factors and the vegetation details of the selected stands of the two forest types are outlined in Table 1.

**Table 1:** Environmental characteristic of the selected stands

Forest types	Selected stands	Dominant plant species	Longitude	Latitude	Elevation (m)
Dry deciduous forest	Sal stand	***Shorea robusta***, *Lagerstroemia parviflora, Diospyros melanoxylon, Terminalia elliptica*	E 083°6′21.2″	N 24°18′59.7″	265
	Sal-mixed stand	***Shorea robusta***, *Anogeissus latifolia, Diospyros melanoxylon, Bridelia retusa, Buchanania lanzan, Hardwickia binata, Mallotus philippensis, Buchanania lanzan*	E 083°6′18.2″	N 24°17′59.7″	258
Temperate forest	Pine stand	***Pinus roxburghii***, *Rhododendron arboretum, Indigofera heterantha, Viburnum cotinifolium*	E 079°32′22.9″	N 29°23′24.4″	1822
	Oak-mixed stand	***Quercus leucotrichophora***, ***Quercus floribunda*** *, Quercus semecarpifolia, Urtica parviflora, Rhododendron arboretum, Berberis asiatica*	E 079°31′43.9″	N 29°21′20.9″	1305
	Oak stand	***Quercus leucotrichophora*** *, Quercus floribunda, Rhododendron arboretum, Myrica esculenta*	E 079°33′08.3″	N 29°21′15.9″	1333

The soil samples were collected within the rhizospheric zone of the existing tree species of selected stands in October 2013, to evaluate the effect of forest types on soil microbial properties. At each selected stand, the samples were taken within 0–10 cm layer after removing the litter as most of the microbial biomass is present in the surface layer. The stands were demarcated into five plots (5 m × 5 m) and the approximate distance in between the plots was about 5 km. Soil samples were collected randomly from six different locations of each plot and a total of thirty samples per stand which were further pooled to form five composite samples per stand. The soil samples were placed in plastic bags and taken to the laboratory. The soil samples were air dried and homogenised manually. Further, the soils were seived using 2 mm mesh to perform physico-chemical analysis. A subsample of each soil was sieved and stored at 4 °C.

### Physico-chemical analysis of soil

Soil pH and electrical conductivity (EC) were obtained using soil:distilled water (1:2.5). Soil texture was done by hydrometric method. Among other parameters water holding capacity (WHC), bulk density (BD), particle density (PD) and porosity were analyzed by the method described by Black et al. (1965). Total organic carbon was estimated using Walkley and Black (1934) method. Available and total nitrogen (AN and TN) was assessed using the specified method given by Stanford and smith (1978) and Kjeldahl nitrogen (Kjeltech analyzer) (Jackson 1958). Available and total phosphorous were estimated by Olsen method (Olsen et al. 1983) and stannous chloride method given by Sparling et al. (1985) followed by hot plate digestion in HNO_3_:HClO_3_ (3:1) at 180 °C for 6 h was used. Available sodium, potassium and calcium concentration were measured using the flame photometric method (systronics-128) (Jackson 1958) followed by extraction with 2 % ammonium acetate buffer. Ammonical nitrogen (NH_4_^+^–N) and nitrate nitrogen (NO_3_^−^–N) were analyzed by Phenate method (APHA 1985) and phenol disulphonic acid method (Jackson 1958), after the extraction of soil samples in KCl and CaSO_4_ solution, respectively.

### Soil microbial biomass C, N and P

Soil microbial biomass carbon (MBC), nitrogen (MBN) and phosphorous (MBP) were estimated using the chloroform fumigation-extraction method given by Brookes et al. (1982, 1985) and Vance et al. (1987) using K_2_SO_4_ (0.5 M) and NaHCO_3_ (0.5 M) as extracting solution in MBC, MBN and MBP, respectively. For MBC, the extract was filtered with Whatman No. 42 filter paper and the filtrate was analyzed by potassium dichromate method explained by Vance et al. (1987). For MBN, the filtrate was processed using Kjeldahl method. Similarly, for MBP, the filtrate was analyzed using Olsen method (Olsen et al. 1983). The final MBC, MBN, and MBP were calculated from differences between fumigated and non-fumigated samples with a conversion factor of 0.33, 0.54 and 0.40 for MBC, MBN and MBP, respectively. Microbial quotient, i.e., the percentage of organic C presently as microbial C is calculated as the ratio of microbial biomass carbon to total organic carbon (MBC/TOC).

### Soil basal respiration

Soil respiration was quantified using alkali absorption method using the moist soil sample. The 25 g of moist soil samples were incubated in a closed jar containing 5 ml of NaOH (0.1 M) in a flask inside it, for 10 days at 25 °C. After incubation the NaOH was titrated with 1 N HCl followed by the addition of 1 ml BaCl_2_ and two drops of phenolphthalein indicator in NaOH. The basal respiration was computed using the difference of the HCl volume used for the titration of sample and the control. The final value was expressed as the amount of CO_2_ evolved from microbes present per gm of soil per hour (µg CO_2_ g^−1^ soil h^−1^). The metabolic quotient (*q*CO_2_) was calculated as the ratio of basal respiration (µg CO_2_ g^−1^ soil h^−1^) to MBC.

### Soil enzymatic assay

The activity of acid phosphatase (EC 3.1.3.2) and β-glucosidase (EC 3.2.1.21) were determined as described by Eivazi and Tabatabai (1977, 1988). *P*-Nitro phenyl phosphate and *p*-nitro phenyl-β-d-glucopyranoside (*p*NPG) was used as a substrate, respectively, and the quantity of p-nitrophenol released during the incubation of per gram sample with substrate at 37 °C for 1 h was measured at 400 nm.

The activity of protease (EC 3.4.21.19) was measured using tyrosine as standard and the amount of amino acids released after incubation of the sample with casein for 2 h at 50 °C was quantified by Ladd and Butler (1972) protocol and the final absorbance were measured at 700 nm.

The dehydrogenase (EC 1.1.1) assay was done using Casida et al. (1964), measured the reduction of 2, 3, 5-triphenyltetrazolium chloride (TTC) to triphenyl formazan on incubation at 30 °C for 24 h and the absorbance were taken at 485 nm.

Fluorescein diacetate (FDA) hydrolysis assay was determined by the method described by Stubberfield and Shaw (1990). In brief, 2-g soil sample was mixed with 15 ml of phosphate buffer (60 mM, pH 7.6) and FDA solution (prepare in acetone), incubated at 30 °C, on an orbital shaker. The reaction was stopped after 20 min by adding chloroform:methanol solution (2:1). Then the soil suspension was centrifuged and absorbance of supernatant was taken at 490 nm.

### Statistical analysis

Data were summarized as mean ± SD (standard deviation). Groups were compared by Student’s *t* test. The groups were also compared by one-way analysis of variance (ANOVA) and the significance of mean difference between the groups was done by Duncan multiple range test (DMRT) and Bonferroni test after adjusting the multiple contrasts. Pearson correlation analysis was done to assess associations between the variables. A two-tailed (*α* = 2) *P* value less than 0.05 (*P* < 0.05) was considered statistically significant. Analyses were performed using SPSS software (version 16.0).

## Results

### Soil physico-chemical characteristics

The physical characteristics of soil of five subgroups (sal, sal-mixed, oak, pine and oak-mixed) and two groups (dry deciduous forest and temperate forest) are summarized in (Tables 2, 3). Among subgroups, the pH of the soils ranged from 6.33 to 6.88. The pH was highest in oak and least at sal soil. The highest EC was recorded in oak soil (265.33). The soil of sal-mixed stand located in the dry deciduous region had the lowest EC value of 31.33. Comparing each physical and chemical characteristic among the subgroups, ANOVA/Bonferroni test revealed significant (*P* < 0.01 or *P* < 0.001) different characteristics among the subgroups. The mean physico-chemical levels of most of the variables were highest at soil of oak-dominated stand.

**Table 2:** Physical characteristics of selected stands

Soil parameters	Dry deciduous forest		Temperate forest		
	Forest stand 1	Forest stand 2	Forest stand 3	Forest stand 4	Forest stand 5
Clay (%)	27.33 ± 1.15 (c)	19.33 ± 1.15 (d)	17.33 ± 1.15 (d)	39.33 ± 1.15 (a)	34.67 ± 1.15 (b)
Slit (%)	6.00 ± 2.00 (d)	20.67 ± 1.15 (a)	9.33 ± 1.15 (c)	8.00 ± 2.00 (cd)	16.67 ± 1.15 (b)
Sand (%)	66.67 ± 1.15 (b)	60.00 ± 0.00 (c)	73.33 ± 1.15 (a)	52.67 ± 1.15 (d)	48.67 ± 1.15 (e)
Bulk density (g cc^−1^)	1.15 ± 0.02 (b)	1.30 ± 0.14 (a)	1.02 ± 0.01 (c)	0.99 ± 0.01 (c)	1.09 ± 0.01 (bc)
Particle density	1.72 ± 0.08 (b)	2.94 ± 0.56 (a)	1.43 ± 0.22 (b)	1.34 ± 0.06 (b)	1.34 ± 0.14 (b)
Porosity (%)	32.89 ± 4.27 (b)	54.07 ± 14.42 (a)	27.72 ± 9.95 (b)	25.79 ± 2.81 (b)	18.19 ± 9.21 (b)
WHC (%)	50.67 ± 3.96 (ab)	45.87 ± 1.87 (b)	53.86 ± 5.27 (a)	48.89 ± 2.33 (ab)	47.05 ± 3.22 (ab)
Moisture (%)	16.99 ± 0.93 (ab)	16.11 ± 0.25 (b)	18.32 ± 1.05 (a)	17.72 ± 0.91 (a)	16.09 ± 0.63 (b)

Values are mean ± SD and letters represent difference significant at *P* < 0.05

*WHC* water holding capacity

**Table 3:** Chemical characteristics of selected stands

Soil parameters	Dry deciduous forest		Temperate forest		
	Forest stand 1	Forest stand 2	Forest stand 3	Forest stand 4	Forest stand 5
pH	6.36 ± 0.04 (d)	6.33 ± 0.01 (c)	6.48 ± 0.02 (b)	6.61 ± 0.05 (b)	6.88 ± 0.02 (a)
EC	83.67 ± 0.91 (d)	31.33 ± 1.09 (e)	109.63 ± 1.06 (c)	190.73 ± 10.36 (b)	265.33 ± 7.74 (a)
TOC (mg g^−1^)	3.71 ± 0.92 (e)	9.02 ± 0.92 (d)	45.11 ± 2.30 (a)	41.79 ± 1.99 (b)	28.39 ± 0.46 (c)
TP (mg g^−1^)	0.26 ± 0.01 (e)	0.29 ± 0.01 (d)	0.32 ± 0.01 (c)	0.37 ± 0.01 (b)	0.56 ± 0.02 (a)
TN (mg g^−1^)	0.64 ± 0.03 (e)	0.75 ± 0.01 (d)	2.14 ± 0.06 (c)	3.16 ± 0.02 (a)	2.27 ± 0.06 (b)
TK (mg g^−1^)	23.26 ± 0.42 (b)	26.67 ± 0.18 (a)	7.44 ± 0.42 (d)	3.64 ± 0.05 (e)	16.04 ± 1.14 (c)
AN (µg g^−1^)	299.52 ± 64.85 (c)	224.64 ± 85.79 (c)	767.52 ± 56.16 (b)	1216.80 ± 112.32 (a)	748.80 ± 85.79 (b)
NH_3_−N (µg g^−1^)	3.50 ± 0.25 (d)	4.87 ± 0.24 (d)	2.85 ± 0.63 (c)	6.54 ± 0.09 (a)	6.62 ± 0.08 (b)
NO_3_–N (µg g^−1^)	2.05 ± 0.81 (d)	1.01 ± 0.47 (c)	1.23 ± 0.78 (e)	6.15 ± 0.34 (b)	6.22 ± 0.56 (a)
AP (µg g^−1^)	39.87 ± 1.87 (c)	42.18 ± 2.55 (c)	37.71 ± 3.88 (c)	134.51 ± 24.28 (b)	201.71 ± 9.73 (a)
ANa (µg g^−1^)	58.05 ± 0.99 (b)	28.24 ± 1.97 (d)	27.47 ± 1.04 (d)	38.19 ± 1.72 (c)	164.72 ± 3.60 (a)
AK (µg g^−1^)	24.37 ± 2.00 (e)	55.76 ± 6.17 (d)	143.28 ± 1.87 (c)	489.09 ± 3.42 (a)	255.79 ± 4.06 (b)
ACa (µg g^−1^)	80.72 ± 3.68 (e)	385.52 ± 3.69 (d)	753.07 ± 1.12 (c)	784.53 ± 5.31 (b)	968.03 ± 6.43 (a)
C/N (µg g^−1^)	5.81 ± 1.24 (c)	11.98 ± 1.29 (b)	21.07 ± 1.66 (a)	13.21 ± 0.61 (b)	12.49 ± 0.38 (b)

Values are mean ± SD and letters represent difference significant at *P* < 0.05

*TOC* total organic carbon, *TP* total phosphorous, *TN* total nitrogen, *TK* total potassium, *AN* available nitrogen, *AP* available phosphorous, *ANa* available sodium, *AK* available potassium, *ACa* available calcium

Further, comparing the mean physical characteristic of each between the two groups,* t* test revealed significant (*P* < 0.05 or *P* < 0.01 or *P* < 0.001) difference. Higher bulk density, particle density and porosity are found in dry deciduous forest as compared to temperate forest while pH and EC were significantly (*P* < 0.01) different and higher in temperate forest as compared to dry deciduous forest. However, some of the characteristics did not differ (*P* > 0.01) between the two groups and were found to be statistically the same.

### Soil microbial biomass C, N and P

**Fig. 2:**
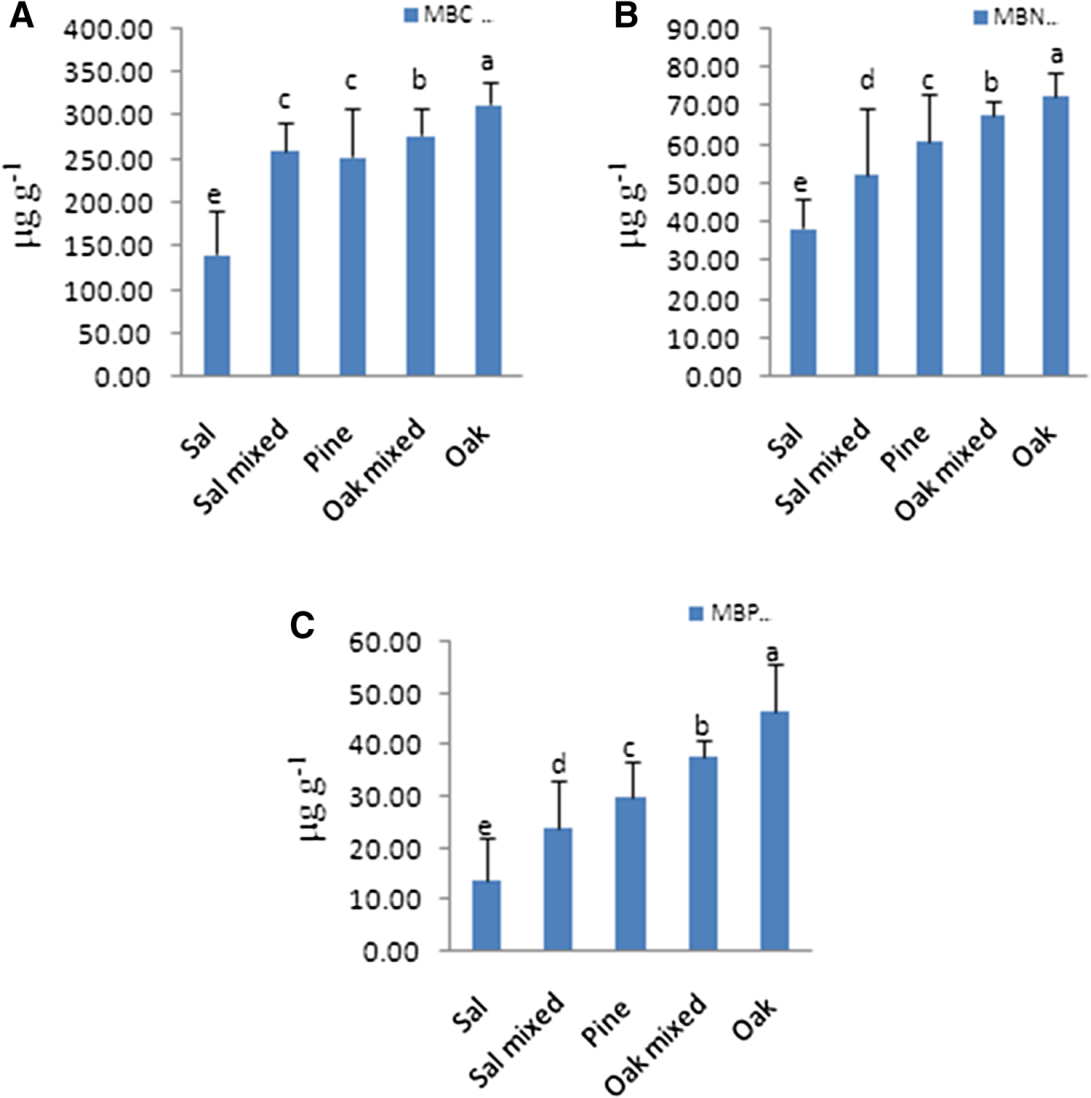
Microbial biomass **a** carbon, **b** nitrogen, **c** phosphorous of soil samples collected from selected stands. Values are expressed as mean ± SD and *letters* represent difference significant at *P* < 0.05

The microbial biomass characteristics of soil of five subgroups and two groups are summarized in (Fig. 2 and Table 4). Soil microbial biomass carbon (MBC) ranged from 140.75 to 313 µg g^−1^. Both the oak-mixed and oak soil had the highest MBC whereas the soil of sal stand had the lowest MBC, i.e., 140 µg g^−1^. The MBC in the case of oak soil was found to be just double of that of the value obtained from the sal soil. In case of MBN, oak soil had the highest MBN and sal soil had the lowest MBN value. The value of MBN varied from 35.55 to 71.35 µg g^−1^. The MBP value was higher in soil of oak-dominated stand, i.e. 46.66 µg g^−1^ and lowest in the soil of sal stand. Statistical analysis revealed significant variations among the soil microbial characteristics. Comparing the mean microbial biomass level of the subgroups, ANOVA/Bonferroni test revealed significantly (*P* < 0.05 or *P* < 0.01 or *P* < 0.001) different levels and the mean microbial biomass levels were highest at soil of oak-dominated stand while least at sal-dominated soil. Similarly, there is significant effect of forest type on soil MBC/TOC, i.e., microbial quotient, MBN/TN and MBN/TP ratios.

**Table 4:** Microbial biomass C, N and P ratio of selected stands

C:N:P ratio	Dry deciduous forest		Temperate forest		
	Forest stand 1	Forest stand 2	Forest stand 3	Forest stand 4	Forest stand 5
BR/MBC (*q*CO_2_)	0.14	0.14	0.10	0.17	0.18
MBC/TOC	3.92	1.44	0.46	0.32	0.36
MBC/MBN	3.89	5.31	4.14	4.08	4.37
MBN/TN (%)	6.03	6.95	2.86	2.15	3.19
MBP/TP (%)	5.36	8.39	9.26	10.11	8.36
MBC/MBP	12.97	12.61	8.58	7.43	6.89

The comparison of mean microbial biomass level of each between the two groups, *t* test revealed significant (*P* < 0.05 or *P* < 0.01 or *P* < 0.001) difference among the two forest type. The overall MBC, MBN, and MBP were recorded higher in temperate as compared to dry deciduous forest.

### Soil basal respiration and metabolic quotient (qCO_2_)

**Fig. 3:**
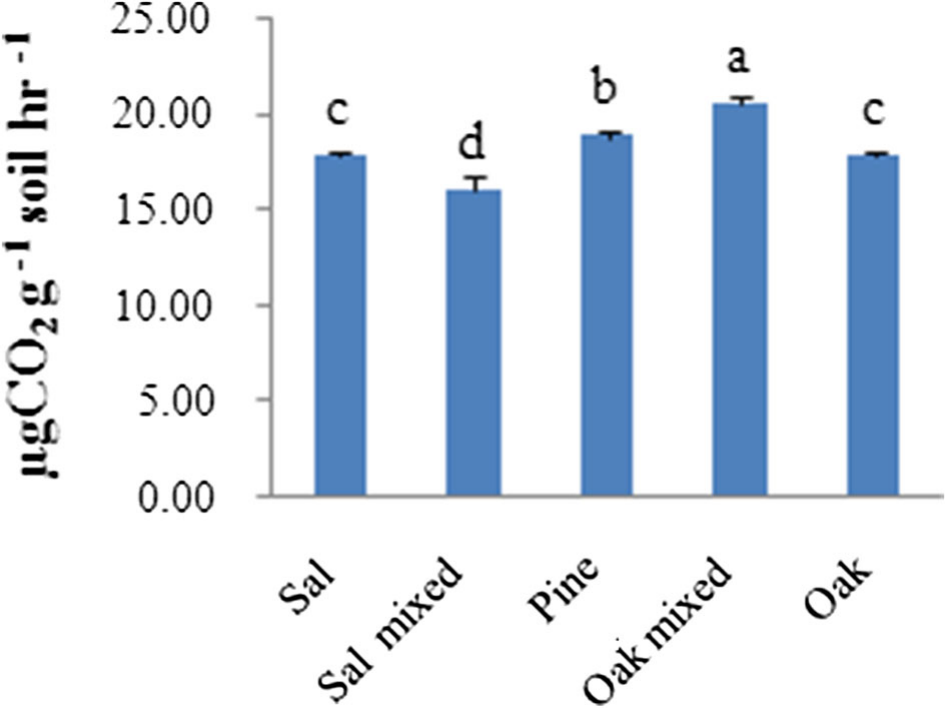
Basal respiration (Bs) in soil collected from selected stands. Values are mean ± SD and *letters* represent difference significant at *P* < 0.05

The basal respiration is high in the soil of oak-mixed stand that was recorded highest among all the forest (Fig. 3); it declined to 16.01 in the sal soil. The variation in the pattern of respiration values is quite low and it ranged from 20.53 µg CO_2_ g^−1^ soil h^−1^ in oak-mixed stand, followed by 18.82 µg CO_2_ g^−1^ soil h^−1^ in pine-dominated stand, 17.84 µg CO_2_ g^−1^ soil h^−1^ in oak-dominated soil which is similar to sal soil and lowest in the soil of sal-mixed stand, whereas the value of metabolic quotient (*q*CO_2_) varied from 0.10 to 0.18. The *q*CO_2_ was high in soil of oak-dominated stand located in temperate region.

Comparing the mean level of soil basal respiration and metabolic quotient (*q*CO_2_) of each among the subgroups, ANOVA/Bonferroni test revealed different levels of significance (*P* < 0.05 or *P* < 0.01 or *P* < 0.001).

Further, comparing the mean respiration level of each between the two groups, t test revealed significant (*P* < 0.05 or *P* < 0.01 or *P* < 0.001) difference among the two forest types and higher respiration rate and metabolic quotient in temperate forest as compared to dry deciduous forest type.

### Enzyme activities

**Fig. 4:**
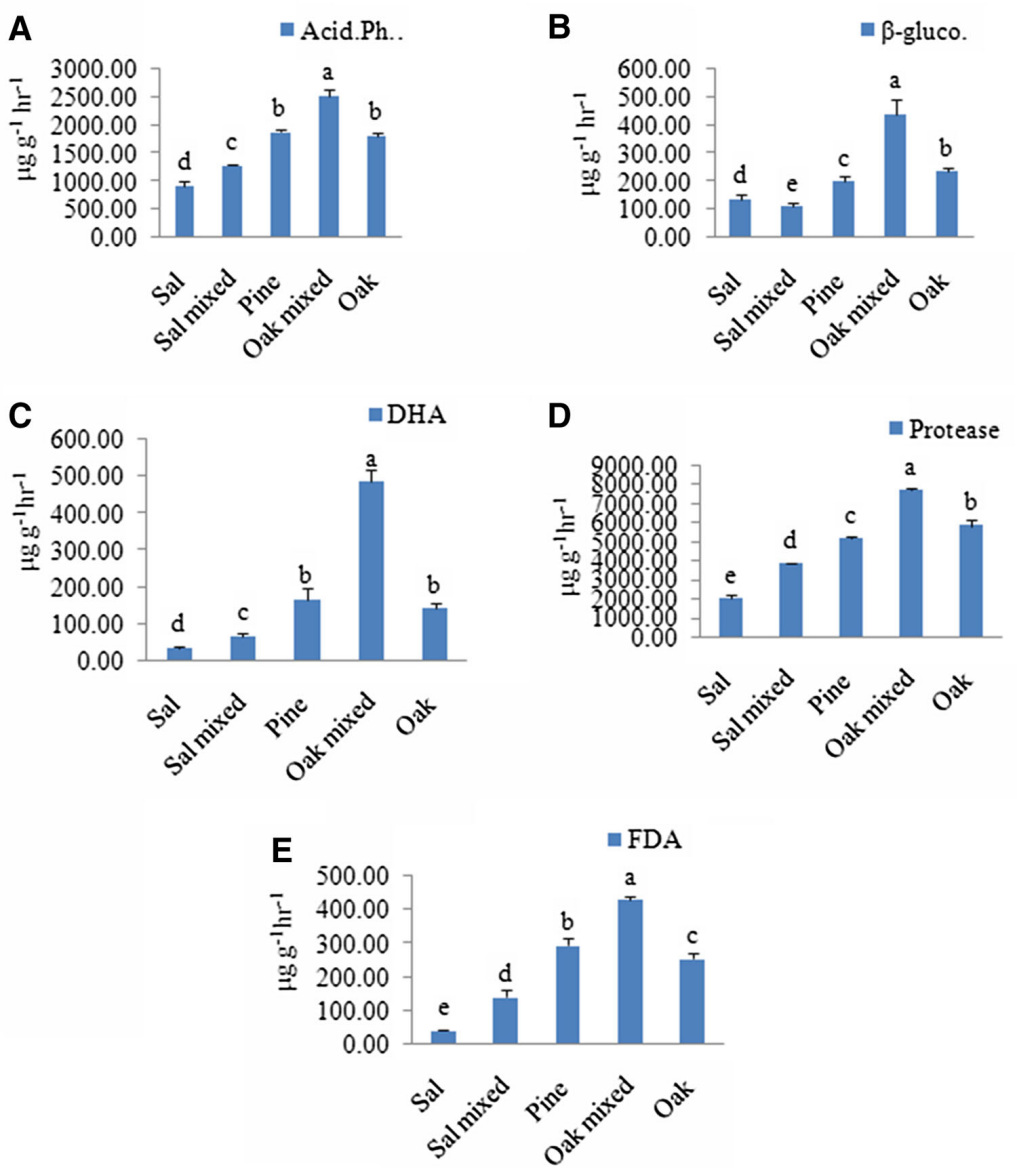
Enzyme activities of soil samples collected from selected stands. **a** Acid phosphatase, **b** β-glucosidase, **c** dehydrogenase, **d** protease, **e** fluorescein diacetate. Values are mean ± SD and *letters* represent difference significant at *P* < 0.05

The enzyme activities of soil of five subgroups are summarized in Fig. 4. The enzymatic activity of selected soil samples were also varied with forest types, which ranging from 929 to 2525.01 µg g^−1^ h^−1^ for acid phosphates, 110.73 to 441.50 µg g^−1^ h^−1^ for β-glucosidase, and DHA varied from 35 to 485.35 µg g^−1^ h^−1^. The value of protease varied from 2018.60 to 7721.07 µg g^−1^ h^−1^ and for FDA it ranged from 41.06 to 429.42 µg g^−1^ h^−1^. Among subgroups, the mean enzyme activities were recorded highest in soil of oak-mixed stand and least mostly at sal soil, and ANOVA/Bonferroni test revealed significantly (*P* < 0.001) different enzyme activities among the subgroups.

Further, *t*test revealed significant (*P* < 0.01 or *P* < 0.001) difference in the enzyme activity of each between the two groups and higher acid phosphatase, β-glucosidase, DHA, protease and FDA activity in temperate forest as compared to dry deciduous forest.

### Pearson’s correlation analysis

Pearson’s correlation revealed that different soil variables were significantly correlated with each other. A diverse range of correlation was recorded among different variable. The pH was positively correlated with biological parameters including TOC, MBC, MBN, MBP, basal respiration and enzymatic activity. Additionally, basal respiration, MBC, MBN, MBP, TOC and activities of DHA and FDA were positively correlated with each other. Similarly, activities of acid phosphates, β-glucosidase and protease were significantly correlated with AP, TP, AN, TN, TOC, NH_4_^+^–N, NO_3_^−^–N, ACa with an exception of TK which was negatively correlated with most of the biological variables and enzymatic activity (Table 5).

**Table 5:** Pearson’s correlation coefficient (*r*) between soil physico-chemical and microbial properties

	Clay	Slit	Sand	pH	EC	BD	PD	Porosity	WHC
Slit	−0.249	1							
Sand	−0.789**	−0.398	1						
pH	0.385	0.146	−0.458	1					
EC	0.105	0.889**	−0.663**	0.281	1				
BD	−0.412	0.563*	0.033	−0.565*	0.469	1			
PD	−0.507	0.591*	0.106	−0.527*	0.502	0.669**	1		
Porosity	−0.492	0.373	0.230	−0.554*	0.307	0.496	0.944**	1	
WHC	−0.243	−0.577*	0.596*	0.000	−0.622*	−0.391	−0.418	−0.345	1
TOC	0.213	−0.243	−0.048	0.849**	−0.144	−0.778**	−0.593*	−0.484	0.288
MBC	0.263	0.465	−0.544*	0.759**	0.582*	−0.227	−0.081	−0.192	−0.307
MBP	0.513	0.205	−0.616*	0.819**	0.402	−0.339	−0.433	−0.440	−0.208
MBN	0.225	0.260	−0.378	0.691**	0.384	−0.350	−0.148	−0.247	0.040
AN	0.628*	−0.371	−0.359	0.772**	−0.100	−0.798**	−0.679**	−0.565*	0.182
TN	0.599*	−0.225	−0.425	0.879**	0.008	−0.772**	−0.667**	−0.602*	0.114
NH3	0.642**	−0.086	−0.553*	0.900**	0.146	−0.696**	−0.612*	−0.575*	−0.030
NO3	0.907**	0.005	−0.862**	0.654**	0.264	−0.447	−0.565*	−0.610*	−0.277
AP	0.795**	0.182	−0.869**	0.705**	0.415	−0.346	−0.466	−0.550*	−0.304
TP	0.563*	0.314	−0.732**	0.778**	0.424	−0.336	−0.450	−0.571*	−0.222
ANa	0.487	0.265	−0.630*	0.402	0.295	−0.086	−0.366	−0.512	−0.250
AK	0.780**	−0.194	−0.615*	0.730**	0.134	−0.636*	−0.533*	−0.478	−0.075
TK	−0.387	0.476	0.064	−0.730**	0.318	0.851**	0.711**	0.573*	−0.346
ACa	0.375	0.200	−0.482	0.994**	0.317	−0.552*	−0.498	−0.533*	−0.028
BR	0.559*	−0.675**	−0.101	0.478	−0.465	−0.853**	−0.734**	−0.547*	0.381
Acidpho	0.519*	−0.145	−0.400	0.850**	0.111	−0.687**	−0.508	−0.439	0.087
Bgluco	0.748**	−0.346	−0.489	0.618*	−0.037	−0.684**	−0.572*	−0.482	0.026
DHA	0.648**	−0.323	−0.409	0.594*	0.002	−0.631*	−0.441	−0.327	0.002
Protease	0.561*	0.016	−0.541*	0.881**	0.265	−0.604*	−0.454	−0.440	−0.053
FDA	0.458	−0.127	−0.353	0.847**	0.099	−0.699**	−0.484	−0.423	0.104

	TOC	MBC	MBP	MBN	AN	TN	NH_3_	NO_3_	AP	TP
Slit										
Sand										
pH										
EC										
BD										
PD										
Porosity										
WHC										
TOC	1									
MBC	0.517*	1								
MBP	0.584*	0.726**	1							
MBN	0.490	0.514	0.399	1						
AN	0.859**	0.448	0.641*	0.456	1					
TN	0.898**	0.579*	0.695**	0.523*	0.967**	1				
NH3	0.834**	0.653**	0.773**	0.516*	0.926**	0.974**	1			
NO3	0.379	0.483	0.698**	0.424	0.660**	0.713**	0.778**	1		
AP	0.335	0.576*	0.769**	0.548*	0.563*	0.629*	0.732**	0.943**	1	
TP	0.391	0.677**	0.805**	0.613*	0.465	0.576*	0.678**	0.818**	0.931**	1
ANa	−0.029	0.338	0.534*	0.378	0.086	0.177	0.281	0.681**	0.806**	0.867**
AK	0.705**	0.521*	0.636*	0.432	0.924**	0.932**	0.928**	0.798**	0.670**	0.510
TK	−0.944**	−0.359	−0.477	−0.356	−0.927**	−0.924**	−0.844**	−0.447	−0.325	−0.294
ACa	0.818**	0.783**	0.838**	0.695**	0.740**	0.849**	0.882**	0.660**	0.728**	0.815**
BR	0.730**	0.069	0.324	0.162	0.893**	0.812**	0.732**	0.494	0.316	0.169
Acidpho	0.891**	0.582*	0.652**	0.553*	0.943**	0.971**	0.937**	0.618*	0.534*	0.470
Bgluco	0.687**	0.365	0.517*	0.339	0.918**	0.895**	0.851**	0.719**	0.534*	0.365
DHA	0.713**	0.404	0.469	0.302	0.903**	0.874**	0.839**	0.592*	0.430	0.242
Protease	0.830**	0.701**	0.705**	0.582*	0.898**	0.955**	0.956**	0.697**	0.634*	0.575*
FDA	0.906**	0.608*	0.610*	0.558*	0.927**	0.963**	0.927**	0.568*	0.487	0.442

	ANa	AK	TK	ACa	BR	Acidpho	Bgluco	DHA	Protease
Slit									
Sand									
pH									
EC									
BD									
PD									
Porosity									
WHC									
TOC									
MBC									
MBP									
MBN									
AN									
TN									
NH3									
NO3									
AP									
TP									
ANa	1								
AK	0.147	1							
TK	0.091	−0.795**	1						
ACa	0.453	0.698**	−0.683**	1					
BR	−0.100	0.781**	−0.897**	0.435	1				
Acidpho	0.023	0.929**	−0.897**	0.814**	0.765**	1			
Bgluco	0.034	0.966**	−0.817**	0.580*	0.846**	0.889**	1		
DHA	−0.149	0.949**	−0.830**	0.545*	0.832**	0.911**	0.941**	1	
Protease	0.133	0.936**	−0.817**	0.858**	0.668**	0.976**	0.867**	0.879**	1
FDA	−0.021	0.905**	−0.904**	0.813**	0.750**	0.992**	0.870**	0.896**	0.974**

* Correlation is significant at the 0.05 level (2-tailed)

** Correlation is significant at the 0.01 level (2-tailed)

## Discussion

The pH of all the soil samples was acidic in nature. The availability of nutrients for plant is highly influenced by soil pH and it indicates the soil fertility (Zhao et al. 2012). The presence of dense fine root on the top layer of soil and higher organic carbon content results in high WHC among the oak-mixed soil and the values were comparable with other results (Paudel and Sah 2003).

Soil microbial biomass indicates the living portion of soil organic matter and is mainly responsible for the conversion of complex into available form of nutrients. The soil microbial status is emphasized by the microbial biomass and activity (Altieri 1999). In the study, the soil with microbial biomass, i.e., MBC, MBN and MBP was extensively found in temperate region indicating the higher availability of nutrients in the respective region and the values were in the range same as recorded by Tripathi and Singh 2013. MBC was observed as positively correlated with soil total organic carbon (TOC) in the study, which is congruent with the findings of Yang et al. 2010; Wang and Wang 2007. TOC, MBC and the enzymatic activity were comparatively low in Sal stand than other stand. The soil organic matter governs its physico-chemical characteristics and provides favorable conditions for the survival of functional groups (Horwath 2005). The lowering in the soil organic matter in sal stand directly depleted the concentration of essential nutrients. Additionally, variation in accessibility of nutrients is explicated by the pattern of forest succession that is responsible for the difference in organic matter content. This eventual difference creates a significant variation in the pattern of total carbon, nitrogen and phosphorous content among the selected stands in the study.

It is observed that microbial C/N is low in temperate region, which is in contrast with the study of Carter (1986). Under different conditions, the comparison between the variations in microbial C/N is not always possible as the mineralization rate may vary with changes in conditions (Brookes et al. 1984; Dalal and Mayer 1987). The high MBC/TOC ratio in dry deciduous forest is evidence of the contribution of MBC to TOC that may be the result of high diversity in organic substrate production from the mixed species present. This ratio is also helpful in evaluation of the soil labile carbon availability as well as fraction of soil recalcitrant organic matter and is also used as an indicator of changes in organic matter due to alterations of soil conditions in future (Cheng et al. 2013). The MBC/TOC ratio or microbial quotient in soil has been used for comparing the soil quality parameter across soils with different organic matter content.

The lower values of MBC/TOC in temperate region indicate the lower accessibility of organic substrates provided by the litter of this region. Similarly MBN/TN and MBP/TP represent the availability of nitrogen and phosphorous. In this study, the highest MBN/TN ratio in dry deciduous forest, dominated by sal plantation represents high mineralization and availability of nitrogen in this region whereas nitrogen availability was found to be low in temperate region (Chen et al. 2005). In addition, the Pearson’s correlation suggests positive correlation between pH and microbial biomass, which supports the microbial growth and activity (Aciego and Brookes 2009). Soil micro flora contains several microorganisms that belong to different groups. C/N ratio for each microbial group is different which ranges from 3 to 5 for bacteria, while for fungi, it ranges from 4 to 15 (Recous and Mary 1990). The MBC/MBN ratio in dry deciduous forest reflects the dominance of bacteria in soil microbial biomass whereas in case of temperate region this ratio indicates the abundance of fungal communities in soil microbial biomass. Moreover, maintenance requirements for microbial cells are high under acidic condition of forest soil. Consequently, production of biomass is reduced that results in low MBC/TOC and MBN/TN ratio.

Estimation of basal respiration highlights the active portion of microbial biomass in soil. The result of this study indicates that the activity of microbes is high in oak-mixed soil located in temperate region, sequentially measures the more decomposition of organic matter in this region. Metabolic quotient expresses the CO_2_ generated by microbial biomass and depends on the stress conditions by different factors (Anderson and Domsch 1990). The lower value of *q*CO_2_ in sal-mixed soil suggests less stress conditions to microorganisms indicating easier carbon utilization with low energy requirement and responsible for stable system in this region (Nsabimana et al. 2004).

Highest enzymatic activities in oak-mixed soil are the measure of high metabolic rate of viable soil microorganisms, which is considered as the reflection of high microbial activity and nutrient availability in this region. The enzymatic activity such as dehydrogenase together with basal respiration, *q*CO_2_ and MBC are the major parameters to influence the metabolic ability and functional quality of soil. In the study, the positive relationship between microbial biomass, soil organic matter and enzyme activities was observed which indicates that the enzymatic activities could be partly endocellular (Arunachalam and Pandey 2003).

From the study, it may conclude that the biochemical as well as microbial properties of soil is significantly affected by forest type. Shifting in tree species will manifest the forest and will also affect the physiochemical as well as the biological properties of soil. Due to the climate change and complexity in soil dynamics, it is difficult to measure the relationship between different soil characters and microbial activates accurately. Although, the present study provides a baseline conclusion about the influence of specific forest type on soil physico-chemical and biological condition.
